# Artificial Intelligence-Guided Bronchoscopy is Superior to Human Expert Instruction for the Performance of Critical-Care Physicians: A Randomized Controlled Trial

**DOI:** 10.1097/CCM.0000000000006629

**Published:** 2025-03-20

**Authors:** Kaladerhan O. Agbontaen, Kristoffer M. Cold, David Woods, Vimal Grover, Hatem Soliman Aboumarie, Sundeep Kaul, Lars Konge, Suveer Singh

**Affiliations:** 1 Chelsea and Westminster Hospital NHS Foundation Trust, London, United Kingdom.; 2 Copenhagen Academy for Medical Education and Simulation (CAMES), University of Copenhagen, Copenhagen, Denmark.; 3 Hammersmith Hospital, Imperial College NHS Trust, London, United Kingdom.; 4 Royal Marsden Hospital NHS Foundation Trust, London, United Kingdom.; 5 Royal Brompton and Harefield Hospitals, Guy’s and St Thomas’ Hospitals, London, United Kingdom.; 6 Imperial College London, APMIC, Faculty of Medicine, London, United Kingdom.

**Keywords:** artificial intelligence, augmented reality, bronchoscopy, critical care, medical education, simulation

## Abstract

**OBJECTIVES:**

Bronchoscopy in the mechanically ventilated patient is an important skill for critical-care physicians. However, training opportunity is heterogenous and limited by infrequent caseload or inadequate instructor feedback for satisfactory competencies. A new artificial intelligence (AI) navigational system using augmented reality – the Ambu Broncho Simulator – can guide bronchoscopy training. Is training with the AI system comparable to bedside, expert tutor instruction in improving bronchoscopy performance?

**DESIGN:**

A nonblinded, parallel group randomized controlled trial was conducted.

**SETTING:**

The study was conducted in a simulated setting at an academic university hospital.

**SUBJECTS:**

Critical-care physicians were invited to take part in the study.

**INTERVENTIONS:**

Forty participants received 30 minutes of bronchoscopy training, either guided by AI only (artificial intelligence group [AIG]) or by expert tutor feedback (expert tutor group [ETG]). All participants performed a final full navigation bronchoscopy performance test and completed a cognitive load questionnaire, the NASA Task Load Index .

**MEASUREMENTS AND MAIN RESULTS:**

Mean intersegmental time (MIT = PT/DC), diagnostic completeness (DC), procedure time (PT), structured progress (SP), and number of segments revisited (SR) were measured. The primary outcome measure assessed was MIT, a measure of bronchoscopic performance efficiency. The secondary outcome measures were DC, PT, SP, and SR. Nineteen participants were randomized to the AIG and 21 participants to the ETG. MIT, PT, and SR were significantly better in the AIG compared to the ETG (median difference, *p*): MIT (–7.9 s, 0.027), PT (–77 s, 0.022), SR (–7 segments, 0.019); all showing moderate effect sizes (0.35, 0.36, and 0.37, respectively) as per Cohen’s classification.

There was no significant difference between the groups for all other final test measures.

**CONCLUSIONS:**

Training using an AI system resulted in faster and more efficient bronchoscopy performance by critical-care physicians when compared to expert human tutor instruction. This could change the future of bronchoscopy training in critical care and warrants validation in patients through clinical studies.

**KEY POINTS**

**Question:** Is training with an AI system that incorporates augmented reality comparable to bedside, expert tutor instruction in improving bronchoscopy performance of critical-care physicians?

**Findings:** In this unique, simulated, randomized controlled trial, those who trained using the AI system demonstrated significantly better post-training bronchoscopy performance when compared to those who received expert tutor instruction. The AI arm demonstrated a mean intersegmental time (procedure time/ number of segments visited) that was 7.9 seconds quicker (*p* = 0.027) and showed a moderate effect size of 0.35 as per Cohen’s classification.

**Meaning:** Training using an AI system resulted in better, faster, and more efficient bronchoscopy performance by critical-care physicians when compared to expert human tutor instruction; this could change the future of bronchoscopy training in critical care and warrants validation in patients through clinical studies.

Flexible bronchoscopy is important for optimal management of the critically ill patient, making bronchoscopic proficiency an essential skillset for the critical-care physician (1, 2). Its use is a standard of care for the mechanically ventilated patient (3) with the indications most commonly being diagnostic or therapeutic. High level competency in critical-care bronchoscopy performance helps improve several procedural outcomes, such as diagnostic yield, number of adverse events, consistency across practitioners, and ultimately patient safety and management.

Within intensive care medicine, there is arguably less opportunity and structure for the attainment of necessary bronchoscopic skills and competencies when compared to pulmonary medicine. The absence of dedicated bronchoscopy lists, the sporadic nature of bronchoscopy necessity in the ICU, and its less traditional seating in the armament of the intensivist all contribute to the issue. The prefellowship resident, or indeed senior resident, if not in a pulmonary critical-care stream, will be subject to this variation in practice and training. A direct consequence of this is that a majority of ICUs still apply the now controversial Halstedian “see-one, do-one, teach-one” paradigm (4) to bronchoscopy training, whereby a training critical-care physician is likely to perform procedures on real patients early in their development of competency. This apprenticeship approach raises patient safety concerns (4, 5), and studies speak of the increased complication rates and suboptimal diagnostic and therapeutic results with novice bronchoscopists (5–7). Dedicated and consistent expert tutor instruction offers knowledge, skills acquisition, and competency assessment, but is time and resource heavy. A reliable adjunct or alternative to expert instructor-delivered feedback and assessment during bronchoscopic training may then be influential for training programs.

Virtual reality (VR) simulation bronchoscopy improves performance (5, 8–14) and provides effective experiential learning opportunities that forego patient risk (15). However, VR does not provide the haptic feedback that practice on a model or human endobronchial tree offers. Now, a novel artificial intelligence (AI) bronchoscopic positioning system exists, that uses augmented reality (AR) to teach bronchoscopy differently to existing instructor delivered-hands-on courses or VR systems – the Ambu Broncho Simulator. Recent studies using this system have already demonstrated its ability to improve the bronchoscopy performance of pulmonary bronchoscopists of all experiences when used (16).

The current study compares guidance and feedback from the AI system to that delivered by expert tutors, for attaining bronchoscopy performance skills in critical-care physicians.

## MATERIALS AND METHODS

### Study Design

A parallel group randomized controlled trial (RCT) of critical-care physicians was conducted in a simulated setting at Imperial College London, Chelsea and Westminster Hospital, United Kingdom. The study conformed to the Consolidated Standards of Reporting Trials guidelines for simulation-based research (17) (**Supplementary Material 1**, http://links.lww.com/CCM/H701). Participants were invited to take part via email and advertisement in national, regional, and local critical-care networks. An online scheduling platform (Calendly; https://calendly.com/) was used to arrange volunteer participation. Those that had performed at least one prior unsupervised bronchoscopy in a critically ill patient on ICU were eligible for study participation. Study information sheets and consent forms were sent out prior to participation. Participants were randomized to the AI training group (AIG) or the expert tutor group (ETG) by KA. Nonblinded, 1:1 stratified block randomization, generated by Sealed Envelope (Sealed Envelope, London, United Kingdom), was used to allocate participants within strata immediately prior to training. Random permuted blocks of two and four were applied to strata by gender (male, female) and bronchoscopy experience (novice, < 10/ intermediate, 10–99/ expert, ≥ 100 bronchoscopies performed).

Procedures were performed on a realistic, silicone-based bronchoscopy phantom lung model (Koken Bronchoscopy Training LM-092, Koken, LTH, Tokyo, Japan) that included representations of quintenary bronchi (**Supplementary Material 2**, http://links.lww.com/CCM/H701). A single-use flexible bronchoscope was used (aScope 5 Broncho HD 5.0/2.2, Ambu, Ballerup, Denmark) to carry out each procedure. The bronchoscope was attached to a transportable monitor (aView 2 Advance, Ambu, Ballerup, Denmark) with its images relayed to a 40-inch TV display which the participants used (**Supplementary Material 3a**, http://links.lww.com/CCM/H701). The AI system (Ambu Broncho Simulator, Prototype version AmbuBPS-trainingGUIDE v.0.0.1, Ambu) was attached to the bronchoscopy setup to allow data capture of all procedures and provide training for participants in the AIG.

Procedure metrics that were measured by the AI system for both groups throughout were explained to participants beforehand:

1. Procedure time (PT): a timer started automatically when the main carina was visualized and ended with extraction of the scope and the “escape” key being pressed on the software keyboard by one of the primary investigators.
2. Diagnostic completeness (DC): measured as the total number of segments entered. Maximum score for DC was 18, allocated as 10 segments in the right lung and eight segments in the left lung (segments 1 + 2 fused and no segment 7).
3. Mean intersegmental time (MIT): calculated as PT/ DC. This was used to provide a surrogate for overall procedural efficiency. Previous studies have shown PT as an indicator of bronchoscopy performance (18). However, this does not always directly correlate with procedural performance (e.g., a quick PT but extremely low DC, i.e., incomplete inspection of all the segments). Lower scores suggest better performance efficiency (i.e., more segments visited in a shorter time).
4. Structured progress (SP): a point is awarded every time a participant visualizes a successive segment in correct ascending order.
5. Segment revisits (SR): measured as the total number of times any of the segments were revisited after already having been visualized and entered.

Participants had no prestudy reading materials or resources. All participants were instructed to perform a pretraining baseline complete bronchoscopy (visualize 10 segments on the right and eight segments on the left) in an ordered manner using as little time as possible. Participants were subsequently given 30 minutes of bronchoscopy training.

**Figure 1.**
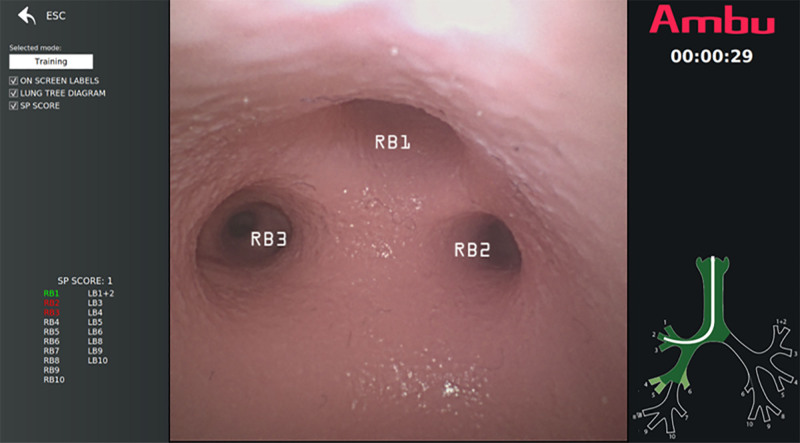
Artificial intelligence training system – the Ambu Broncho Simulator. **Lung tree diagram** (*bottom-right corner*): indicates which branches have been fully visualized (*dark green*) and which have been in view (*light green*) during the examination. The white curvilinear structure indicates the location of the bronchoscope. **Procedure time** (*top-right corner*): starts automatically once the main carina has been visualized. **User training-assistance interface** (*top-left corner*): enables users to select the amount of AI assistance provided by ticking or unticking the checkboxes. **SP (structured progress) score** (*bottom-left corner*): structured progress score and indication of which segments were entered sequentially so giving points (*colored green*), and which segments were entered but out of sequence scoring no SP points (*colored red*). For example, in this image the initial user examination sequence was: main carina -> RB1 -> RB3 -> RB2, giving a score of 1 point. **Screen labels** (*center*): overlayed white anatomical location labels identifying segments in view. The labels turn green once the segment has been entered and remain green for the duration of the examination.

Those randomized to the AIG undertook self-directed training using the AI system (**Supplementary Material 3b**, http://links.lww.com/CCM/H701). The AI system provided feedback using three features which are overlayed in real-time on the screen as a bronchoscopy is carried out (**Fig. 1**):

1. *On-screen labels*–display the names of visible bronchi down to the segmental level. The labels conform to the Yamashita naming system (19), where RB2 is the posterior upper lobe segment. These labels are overlayed on the bronchi and segments in an AR fashion. Once a segment is entered the label turns from white to green.
2. *Lung tree diagram*–displayed in the bottom right corner of the screen, providing information of the bronchoscope’s position within the lung tree. The lung tree areas turn light green when the bronchi are first visualized at a distance and change to dark green when the individual segment has been entered.
3. *SP score*–displayed on the left-hand side of the screen with all the segments listed in white font. Points are given when a bronchial segment is visited in ascending sequential order, beginning from the carina. The listed segment turns from white to green if entered in the correct sequence. If entered out of sequence the segment font turns red. For example, progress of a bronchoscopy in the following order: main carina -> RB1 -> RB2 -> RB3, would score 3 points. Exploration in the order: main carina -> RB3 -> RB1 -> RB2, would score 1 point (20).

Users of the AI system could select the number of the three feedback features active during their training. At the end of each practice run, AIG participants received automated feedback on the monitor, detailing their PT, DC, and SP score. AIG participants were reminded that the final test would be without any assistance from the AI and so were encouraged to have at least one test run with all AI feedback features switched off. The primary investigators observed the AIG during training, not providing any feedback and only being on standby for any AI system issues.

Those randomized to the ETG received one-on-one tutelage from either of two expert pulmonary and critical-care consultants, with a combined experience of over 30 years performing and teaching bronchoscopy (> 6500 bronchoscopies). Both groups were provided with an A3-sized bronchial tree diagram with labeled segmental anatomy to use as desired only during the training period. The ETG participants received no AI feedback during training, only receiving their AI measured performance metrics after their final test bronchoscopy. Feedback on performance during their training was based on comments and guidance from their expert tutor (Supplementary Material 3a, http://links.lww.com/CCM/H701).

Both groups were tasked with training toward achieving the best score possible in PT, DC, SP, and SR in their post-training final test. Both groups used the same equipment. At the end of 30 minutes of training both groups performed a final test bronchoscopy with no AI or expert tutor assistance. The study flow diagram is shown in **Supplementary Material 4** (http://links.lww.com/CCM/H701).

After the final test all participants completed a perceived-workload assessment tool – the NASA Task Load Index (NASA-TLX) (21). This provides information regarding a participant’s task specific cognitive load in domains of mental demand, physical demand, temporal demand, effort, performance, and frustration (21) (**Supplementary Material 5**, http://links.lww.com/CCM/H701).

The AI system automatically recorded and rated all procedures, providing the data used for analysis and comparison of pre- and post-training bronchoscopy performance. The AI system has been validated as able to assess bronchoscopy performance as well as expert human raters (22).

Data collection occurred during a period of 5 days, Monday–Friday, December 4–8, 2023. All participants gave verbal and written informed consent prior to the study and provided their demographic information (**Table 1**). Ethical approval is not required for this simulated study that does not involve patients, as per our local and national guidelines.

**TABLE 1.** Participant Demographics

Demographic	Group	Expert Tutor Group	Artificial Intelligence Group	*p*
Participant number^a^(female sex)	Total	21 (8)	19 (6)	***p* = 0.67** ^a^
	Novice	6 (3)	6 (3)	
	Intermediate	12 (5)	9 (3)	
	Expert	3 (0)	4 (0)	
Age, yr	Total	35.7 ± 6.5	37.3 ± 10.5	***p* = 0.561**
	Novice	30.7 ± 3.4	32.7 ± 11.0	
	Intermediate	36.7 ± 6.6	36.9 ± 7.5	
	Expert	42.0 ± 3.5	45.3 ± 13.5	
Bronchoscopies performed	Total	75.1 ± 215.0	98.0 ± 236.4	***p* = 0.751**
	Novice	2.3 ± 1.4	2.0 ± 0.6	
	Intermediate	26.1 ± 14.8	27.7 ± 13.7	
	Expert	416.7 ± 505.8	400.0 ± 424.3	
Bronchoscopies performed within the last 6 mo	Total	23.4 ± 86.4	**6.3 ± 9.1**	***p* = 0.397**
	Novice	1.7 ± 1.9	1.3 ± 1.2	
	Intermediate	4.7 ± 2.9	5.9 ±7.0	
	Expert	141.7 ± 223.7	14.8 ± 14.6	

a Data are presented as total number (number of female sex) and compared using a chi-square test.

Values are presented as mean ± sd if not otherwise indicated and compared using the independent-samples *t* test. *p* values in boldface font < 0.05 are considered significant.

### Outcome Measures

The primary outcome measure assessed was MIT (PT/DC). The secondary outcomes were PT, DC, SP, and SR.

Based on previous studies using the AI system to train and test bronchoscopic performance (18), the ability to detect a statistically significant difference in PT or DC with 80% power at the alpha 0.05 level was estimated to require 20 participants per group (**Supplementary Material 6**, http://links.lww.com/CCM/H701).

### Statistical Analysis

All data were analyzed using the Statistical Package for the Social Sciences version 29 (PASW v29.0; IBM Corp, Armonk, NY). Histogram, Q-Q plot, and Shapiro-Wilk test analyses suggested all outcome measure data should be interrogated as nonnormally distributed. Therefore, MIT, PT, DC, SP, and SR are reported as median and interquartile range and nonparametric testing is used to compare groups – Mann-Whitney *U* test for independent samples and Wilcoxon signed rank test for paired observations from the same participants at different time points. Mann-Whitney *U* testing was used for comparison of NASA-TLX scores.

## RESULTS

### Baseline Characteristics

Forty participants were voluntarily recruited to the study with 21 assigned to the ETG and 19 assigned to the AIG. There were no significant differences between the groups in allocation of sex, age, or prior bronchoscopy experience (Table 1).

### Final Bronchoscopy Test Performance

Both groups demonstrated significant improvements in pre- to post-training test scores (**Table 2**).

**TABLE 2.** Differences in Pre- and Post-Training Performance for Both Groups – Outliers Included

Group	Metric	Pretraining	Post-Training	*p*
Expert tutor group (*n* = 21)	MIT	52.3	24.4	**< 0.001**
	PT	521	341	**0.011**
	DC	10	16	**< 0.001**
	SP	2	6	**0.002**
	SR	14	11	0.153
Artificial intelligence group (*n* = 19)	MIT	34.3	16.5	**< 0.001**
	PT	380	264	**< 0.001**
	DC	11	16	**< 0.001**
	SP	2	6	**< 0.001**
	SR	8	7	0.407

DC = diagnostic completeness (number of segments visited), MIT = mean intersegmental time (PT/DC in s), PT = procedure time (s), SP = structured progress (points), SR = segment revisits (number of segments).

*p* values are calculated using the Wilcoxon signed rank test. *p* values in boldface font < 0.05 are considered significant.

**Figure 2.**
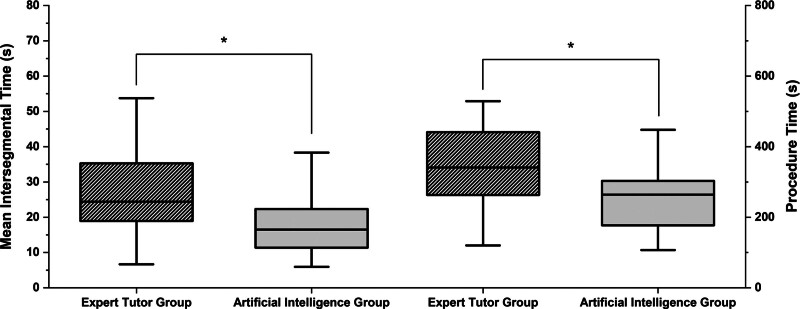
Post-training scores between the expert tutor group and artificial intelligence group compared for mean intersegmental time and procedure time. Mean intersegmental time: Expert tutor group (ETG) (24 s ± 21), artificial intelligence group (AIG) (16.5 s ± 11). Procedure time: ETG (341 s ± 205), AIG (264 s ± 26). Data reported as median ± interquartile range. *p < 0.05 and considered significant.

At final test bronchoscopy, the AIG were (median difference, *p*): more efficient, MIT (–7.9 s, 0.027) and SR (–7 revisits, 0.019), and significantly faster than ETG (–77 s, 0.022) (**Fig. 2**; and **Supplementary Material 7**, http://links.lww.com/CCM/H701). The differences in MIT, PT, and SR had a moderate effect size (0.35, 0.36, and 0.37, respectively) as per Cohen’s classification.

There was no difference between the groups for DC (0 segments, 0.668) and SP (0 points, 0.376) (Supplementary Material 7, http://links.lww.com/CCM/H701).

A secondary analysis was conducted because of significant differences in the baseline pretraining scores between the groups (**Supplementary Material 8**, http://links.lww.com/CCM/H701). Three participants (numbers 23, 31, and 35) from the ETG arm were excluded as outliers as their pretraining PT scores were more than 1.5 (×) the interquartile range greater than the third quartile of the group data. The pretraining PT scores of these outliers were significantly worse than those reported in previous studies, including one testing novice medical students with no prior bronchoscopy experience (18). With outliers removed there were no significant differences between the groups in baseline bronchoscopy performance for PT, DC, MIT, and SP (**Supplementary Material 9**, http://links.lww.com/CCM/H701). The secondary analysis without outliers demonstrated results similar to our primary analysis (**Table 3**; and **Supplementary Material 10**, http://links.lww.com/CCM/H701), showing the AIG post-training performance to be significantly more efficient and faster than the ETG (median difference, *p*): MIT (–7.4 s, 0.049) and PT (–74 s, 0.039) (**Supplementary Material 11**, http://links.lww.com/CCM/H701).

**TABLE 3.** Comparison of Post-Training Final Test Bronchoscopy Performance Between the Expert Tutor Group and Artificial Intelligence Group – Outliers Removed

Metric	Expert Tutor Group (*n* = 18)	Artificial Intelligence Group (*n* = 19)	Difference in Medians	*p*
Mean intersegmental time	23.9	16.5	–7.4	**0.049**
Procedure time	338	264	–74	**0.039**
Diagnostic completeness	15.5	16	+0.5	0.641
Structured progress	5.5	6	+0.5	0.284
Segment revisits	11.5	7	-4.5	0.245

Post-training bronchoscopy performance scores for mean intersegmental time and procedure time were significantly better in the artificial intelligence group arm. There were no significant differences in post-training scores for diagnostic completeness, structured progress, and segment revisits between the two groups. *p* values are calculated using the Mann-Whitney *U* test. *p* values in boldface font < 0.05 are considered significant.

Participant allocation numbers and pre- and post-training performance test results are shown in **Supplementary Materials 12–14** (http://links.lww.com/CCM/H701). Prior bronchoscopy experience for individual clinicians from both groups is shown in **Supplementary Materials 15** and **16** (http://links.lww.com/CCM/H701).

### Perceived Cognitive Load Measure

**Figure 3.**
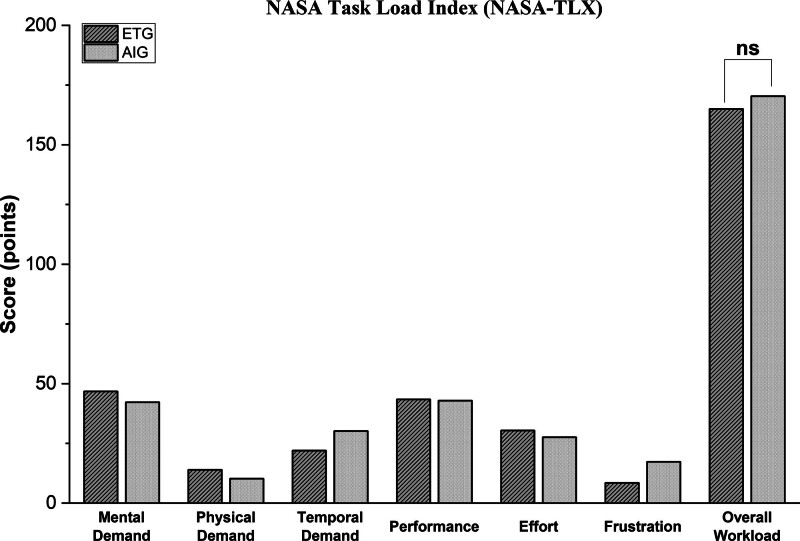
NASA Task Load Index cognitive workload score comparison between expert tutor group and artificial intelligence group – outliers included. *Bar graph* comparing the individual and overall cognitive workload domain scoring of the expert tutor group and artificial intelligence group arms. No significant differences were found in any of the domains between the groups. NS = nonsignificant.

No significant differences were found between the groups for overall perceived workload (median difference, *p*) (+6 points, 0.538) and subgroup measures of workload (**Fig. 3**; and **Supplementary Materials 17** and** 18**, http://links.lww.com/CCM/H701) on NASA-TLX scoring.

## DISCUSSION

This study is the first to compare bronchoscopy training with a novel AI system using AR to that of the current accepted gold standard of bedside expert tutor guided instruction for physicians. Thirty minutes of self-directed, continuous training with the AI system and immediate AI feedback resulted in significantly improved bronchoscopy performance when compared to the ETG. Although both training methods improved bronchoscopy performance (as any training period would be expected to), the AIG arm demonstrated significantly faster procedure (PT) and MITs, proving to be quicker and more efficient than those trained in the ETG. Procedure efficiency and PT are important factors when performing bronchoscopy in the mechanically ventilated patient, as a longer PT increases the propensity for deterioration caused by hypoxemia or other physiologic instability (23, 24). Furthermore, the self-guided nature of the training in the AIG caused no additional participant psychologic stress as evidenced by similar NASA-TLX scoring. This is a novel finding with potentially groundbreaking future implications on bronchoscopy training, especially for critical-care physicians.

How might an automated system lead to better performance than the accepted standard of expert tutor instruction in certain domains? The nonverbal, immediate feedback provided may allow AIG participants better focus while practicing (25), eliminating the potential verbal distraction provided by in-person tutelage. Furthermore, performance feedback without the risk of possible perceived judgment by a human teacher may have provided added psychological safety, better optimizing learning, and subsequent performance of the participant (26). Studies show that the psychological safety provided through learning by VR simulation provides a flow state that leads to better learning outcomes (27). On reflection, this study may have failed to demonstrate significant differences in cognitive workload between the two groups because of the timing of deployment of the NASA-TLX questionnaire. The scores possibly demonstrated the perception of having to do the common-to-both-groups “final test.” Deploying the questionnaire at the end of the training periods (before the final test) would give better insight into cognitive load differences between the styles of instruction (AIG vs. ETG). Additional qualitative studies are needed to fully explore the perceived benefits to learners of this self-directed learning with the AR AI-feedback system.

The unlimited and repetitive procedural practice with instant feedback that the AI system used in our study provides allows users to implement deliberate practice and mastery learning independently for bronchoscopy skill acquisition. This approach to learning shows particular value in acquiring ICU-based procedural skills (28–30) and is associated with better patient outcomes (31–34). It can be argued that the 30 minutes of dedicated, one-on-one bronchoscopy training that the ETG arm received is extremely difficult to consistently replicate in the real-world ICU environment because of competing work pressures. Significant time demands exerted by high volume workload and high acuity patients (35–39) largely makes consistently delivered training akin to the ETG arm impractical, giving a more heterogenous delivery of bronchoscopy training. The availability of an AI training system that can match and supersede the bronchoscopy performance improvement provided by the scarce “gold-standard” resource of one-on-one tutoring is a major benefit of such a system. The expert clinical tutor might then be freed to complement learner training, for instance in improving handling, dexterity and other value based bronchoscopic skills and competencies. Indeed, Jiang et al demonstrated the benefits of teaching fibreoptic intubation using expert-guided practice on a VR simulator (12). In another VR bronchoscopy training study (5), participants gave feedback asking for use of the simulated system but with a human tutor to facilitate. The AI system’s use of AR may very well expand an instructor’s teaching ability. The on-screen overlay labels and lung-tree map provide clearly visible reference points and symbols for both teacher and learner that are not available in the real-world, making the abstract more concrete and allowing easier description during teaching, reducing misunderstanding and improving communication. Future studies should explore how expert tutor facilitated training using the AI system compares to the methods tested in this study for improving bronchoscopy performance.

The AI system in this study additionally possesses the potential for wider applications in physician training. A previous study has shown the ability of this AI system to be used effectively in both a mastery and self-directed approach to learning bronchoscopy (40). Furthermore, VR systems have previously been shown to be as good as bronchoscopy experts for assessing performance (11), and this particular system has been validated as being as good as human raters in assessing bronchoscopy competence (22). This raises the intriguing prospect of using the Ambu Broncho Simulator as a standalone training system that can be used to remotely assess self-directed learning and demonstration of bronchoscopy competencies in physicians. This would have a significant impact on the curriculum for bronchoscopy training standards in pulmonology and intensive care medicine.

The study has several strengths. First, the RCT design is crucial to understanding the added value of such a system against best practice, expert tutor guided instruction. Second, the AR system interacts with a physical phantom lung model, ensuring users receive haptic feedback, such as dynamic resistance to movement of the bronchoscope, providing a simulation experience that is superior to other virtual bronchoscopy simulators and mirrors the development of surgical practice training more generally (41). Third, the AI system software recorded all bronchoscopy performance metrics, removing the potential for human error, providing accurate, consistent, and standardized feedback (8, 14, 22).

There are limitations to this study. First, although the study size is comparable to prior studies in this field and was powered for the outcomes, it was not large enough to allow subgroup analyses, such as analyzing the effect of prior bronchoscopy experience on the impact of bronchoscopy performance in the two groups (39). Second, three outliers were removed from the ETG arm for a secondary analysis because their very poor pretraining test performance metrics led to significant differences in pretraining bronchoscopy performance metrics between the groups (Table 1), despite appropriate randomization. Review of video performance of the participants revealed no measurement, data entry, or processing errors. A feasible explanation could be that part of our stratification for randomization was based on assigning bronchoscopy-experience based purely on “number of bronchoscopies performed.” Indeed, it is worth noting that this is the only study to-date which objectively measured pretraining baseline performance before intervention. This may prove to be a more useful substitute metric for “bronchoscopy experience” in future training studies and further highlights the questionable utility of numbers-based targets for competency (2). Third, the AI system does not provide feedback on aspects of dexterity, such as wall collision and luminal centrality. These performance metrics are available in certain VR platforms (42, 43) and were they to be incorporated into this AI system would reduce the risks of gamification-induced recklessness and further improve the system’s fidelity. Finally, future studies should address the impact of this AI system on bronchoscopy skill retention.

## CONCLUSIONS

An AI bronchoscopic positioning and feedback system using AR (the Ambu Broncho Simulator) was superior to expert tutoring in improving bronchoscopy performance efficiency (superior MIT and SR) and procedural time in critical-care physicians. This could have a significant beneficial impact on curriculum training and assessment of bronchoscopy skills. It’s transferability and impact on patients require urgent evaluation.

## ACKNOWLEDGMENT

We would like to thank Ambu for allowing us to use their Broncho Simulator for this study.

## Supplementary Material


