# Supplementary material for: Artificial Intelligence-Guided Bronchoscopy is Superior to Human Expert Instruction for the Performance of Critical-Care Physicians: A Randomized Controlled Trial
Source: Crit Care Med. 2025 Mar 20;53(5):e1105–15. doi: 10.1097/CCM.0000000000006629 (PMC12047642; doi:10.1097/CCM.0000000000006629)
Supplement: Supplementary file 1 [file ccm-53-e1105-s001.pdf]

SUPPLEMENTARY MATERIALS

Table of Contents

Supplementary Material 1 - CONSORT Checklist 4

Supplementary Material 2 – Koken™ Bronchoscopy Training Model LM-092, Koken CO., LTH, Tokyo, Japan 10

Supplementary Material 3 – Study set up of the Artificial Intelligence system 11

Supplementary Material 4 – Study flow diagram 13

Supplementary Material 5 - NASA-TLX scoring calculation process 14

Supplementary Material 6 – Power and Sample size calculations 19

Supplementary Material 7 – Table: Comparison of post-training median scores between both groups – outliers included 21

Supplementary Material 8 – Table: Comparison of pre-training test median scores between the expert-tutor group (ETG) and artificial-intelligence group (AIG) – outliers included 22

Supplementary Material 9 – Table: Comparison of pre-training median scores between the expert-tutor group (ETG) and artificial-intelligence group (AIG) – outliers removed .23

Supplementary Material 10 – Table: Differences in pre- and post-training performance for both the expert-tutor group (ETG) and artificial-intelligence group (AIG) – outliers removed 24

Supplementary Material 11 – Figure: Post-training scores between ETG and AIG compared for procedural time (PT) and mean intersegmental time (MIT) – outliers removed 25

Supplementary Material 12 - ETG arm participant allocation numbers and bronchoscopy experience – outliers included 26

Supplementary Material 13 – AIG arm participant allocation numbers and bronchoscopy experience – outliers included 28

Supplementary Material 14 – Individual pre- and post-training scores for both the ETG and AIG arms – outliers included 30

Supplementary Material 15 – ETG arm participant bronchoscopy experience 34

Supplementary Material 16 – AIG arm participant bronchoscopy experience 35

Supplementary Material 17 – Table: NASA-TLX cognitive workload scores between the expert-tutor group (ETG) and artificial-intelligence group (AIG) – outliers included 36

Supplementary Material 18 – NASA-TLX cognitive workload score comparison between the expert-tutor group (ETG) and artificial-intelligence group (AIG) – outliers removed .37

Supplementary Material 1 - CONSORT Checklist

Reporting checklist for randomised trial.

Based on the CONSORT guidelines.

		Reporting Item	Page Number
**Title and Abstract**			
**Title**	#1a	Identification as a randomized trial in the title.	**1**
**Abstract**	#1b	Structured summary of trial design, methods, results, and conclusions	**3, 4**
**Introduction**			
**Background and objectives**	#2a	Scientific background and explanation of rationale	**6, 7**
**Background and objectives**	#2b	Specific objectives or hypothesis	**6, 7**
**Methods**			
**Trial design**	#3a	Description of trial design (such as parallel, factorial) including allocation ratio.	**7, 8**
**Trial design**	#3b	Important changes to methods after trial commencement (such as eligibility criteria), with reasons	**N/A**

**Participants**	#4a	Eligibility criteria for participants	**7**
**Participants**	#4b	Settings and locations where the data were collected	**7**
**Interventions**	#5	The experimental and control interventions for each group with sufficient details to allow replication, including how and when they were actually administered	**9, 10**
**Outcomes**	#6a	Completely defined prespecified primary and secondary outcome measures, including how and when they were assessed	**11**
**Outcomes**	#6b	Any changes to trial outcomes after the trial commenced, with reasons	**N/A**
**Sample size**	#7a	How sample size was determined.	**11**
**Sample size**	#7b	When applicable, explanation of any interim analyses and stopping guidelines	**N/A**
**Randomization – Sequence generation**	#8a	Method used to generate the random allocation sequence.	**7**

**Randomization - Sequence generation**	#8b	Type of randomization; details of any restriction (such as blocking and block size)	**7**
**Randomization - Allocation concealment mechanism**	#9	Mechanism used to implement the random allocation sequence (such as sequentially numbered containers), describing any steps taken to conceal the sequence until interventions were assigned	**7**
**Randomization - Implementation**	#10	Who generated the allocation sequence, who enrolled participants, and who assigned participants to interventions	**7**
**Blinding**	#11a	If done, who was blinded after assignment to interventions (for example, participants, care providers, those assessing outcomes) and how.	**N/A**
**Blinding**	#11b	If relevant, description of the similarity of interventions	**N/A**
**Statistical methods**	#12a	Statistical methods used to compare groups for primary and secondary outcomes	**11**

**Statistical methods**	#12b	Methods for additional analyses, such as subgroup analyses and adjusted analyses	**11**
**Results**			
**Participant flow diagram (strongly recommended)**	#13a	For each group, the numbers of participants who were randomly assigned, received intended treatment, and were analysed for the primary outcome	**11, 12, Supplementary Material 4**
**Participant flow**	#13b	For each group, losses and exclusions after randomization, together with reason	**12, Supplementary Material 4**
**Recruitment**	#14a	Dates defining the periods of recruitment and follow-up	**11**
**Recruitment**	#14b	Why the trial ended or was stopped	**N/A**
**Baseline data**	#15	A table showing baseline demographic and clinical characteristics for each group	**Table 1**
**Numbers analysed**	#16	For each group, number of participants (denominator) included in each analysis and whether the analysis was by original assigned groups	**11, 12, 13**

**Outcomes and estimation**	#17a	For each primary and secondary outcome, results for each group, and the estimated effect size and its precision (such as 95% confidence interval)	**12, 13**
**Outcomes and estimation**	#17b	For binary outcomes, presentation of both absolute and relative effect sizes is recommended	**N/A**
**Ancillary analyses**	#18	Results of any other analyses performed, including subgroup analyses and adjusted analyses, distinguishing pre-specified from exploratory	**12, 13**
**Harms**	#19	All important harms or unintended effects in each group (For specific guidance see CONSORT for harms)	**N/A**
**Discussion**			
**Limitations**	#20	Trial limitations, addressing sources of potential bias, imprecision, and, if relevant, multiplicity of analyses	**15, 16**
**Generalisability**	#21	Generalisability (external validity, applicability) of the trial findings	**15, 16**

**Interpretation**	#22	Interpretation consistent with results, balancing benefits and harms, and considering other relevant evidence	**13, 14, 15, 16**
**Registration**	#23	Registration number and name of trial registry	**N/A**
**Protocol**	#24	Where the full trial protocol can be accessed, if available	**N/A**
**Funding**	**#25**	Sources of funding and other support (such as supply of drugs), role of funders	**18**

Supplementary Material 2 – Koken™ Bronchoscopy Training Model LM-092, Koken CO., LTH, Tokyo, Japan

The realistic, silicone-based bronchoscopy phantom lung model that includes representations of quintenary bronchi.

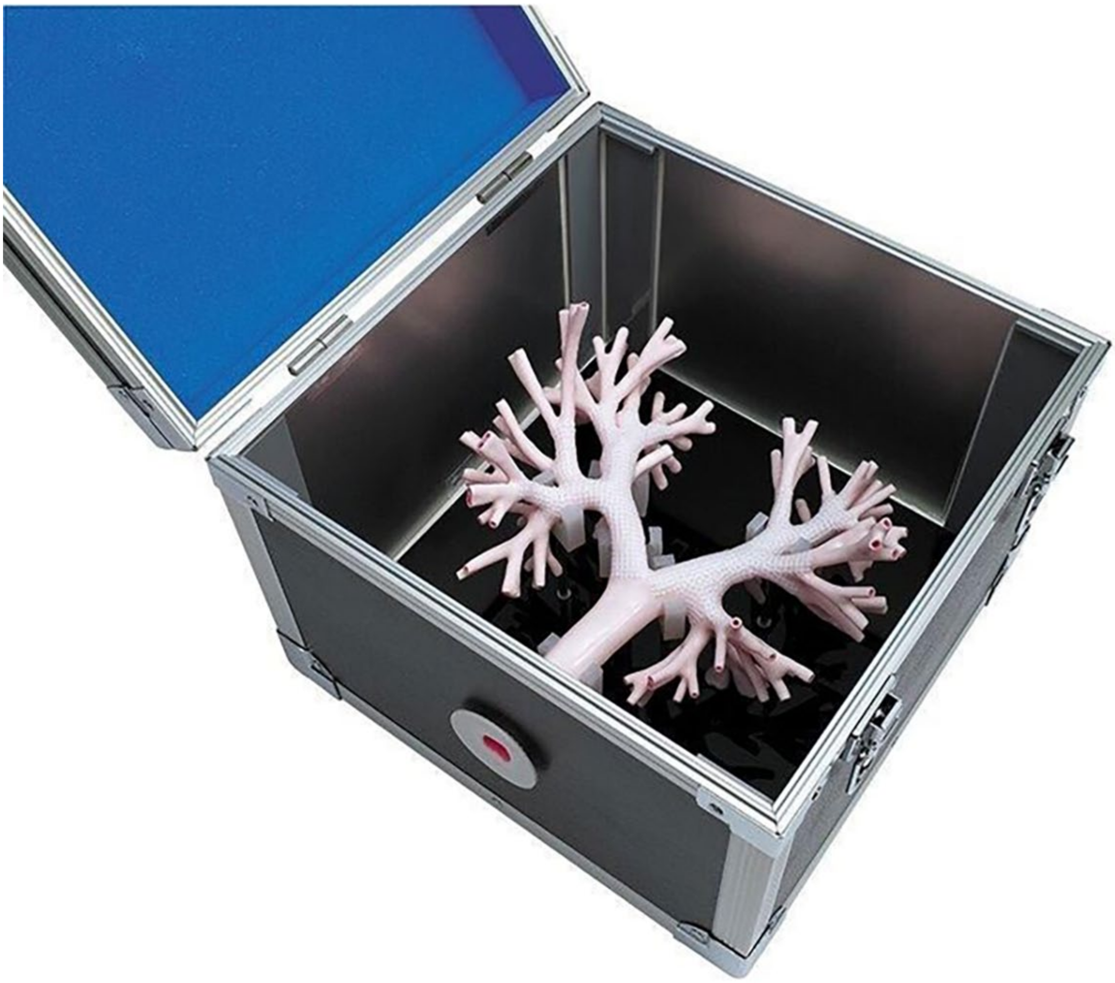

Supplementary Material 3 – Study set up of the Artificial Intelligence system

(a) Participant randomised to the ETG arm with the expert teacher SS (on the right) about to start the software so that it can record the participant's bronchoscopy performance metrics in the background. No artificial intelligence feedback was visible to the participant until after their final post-training test.

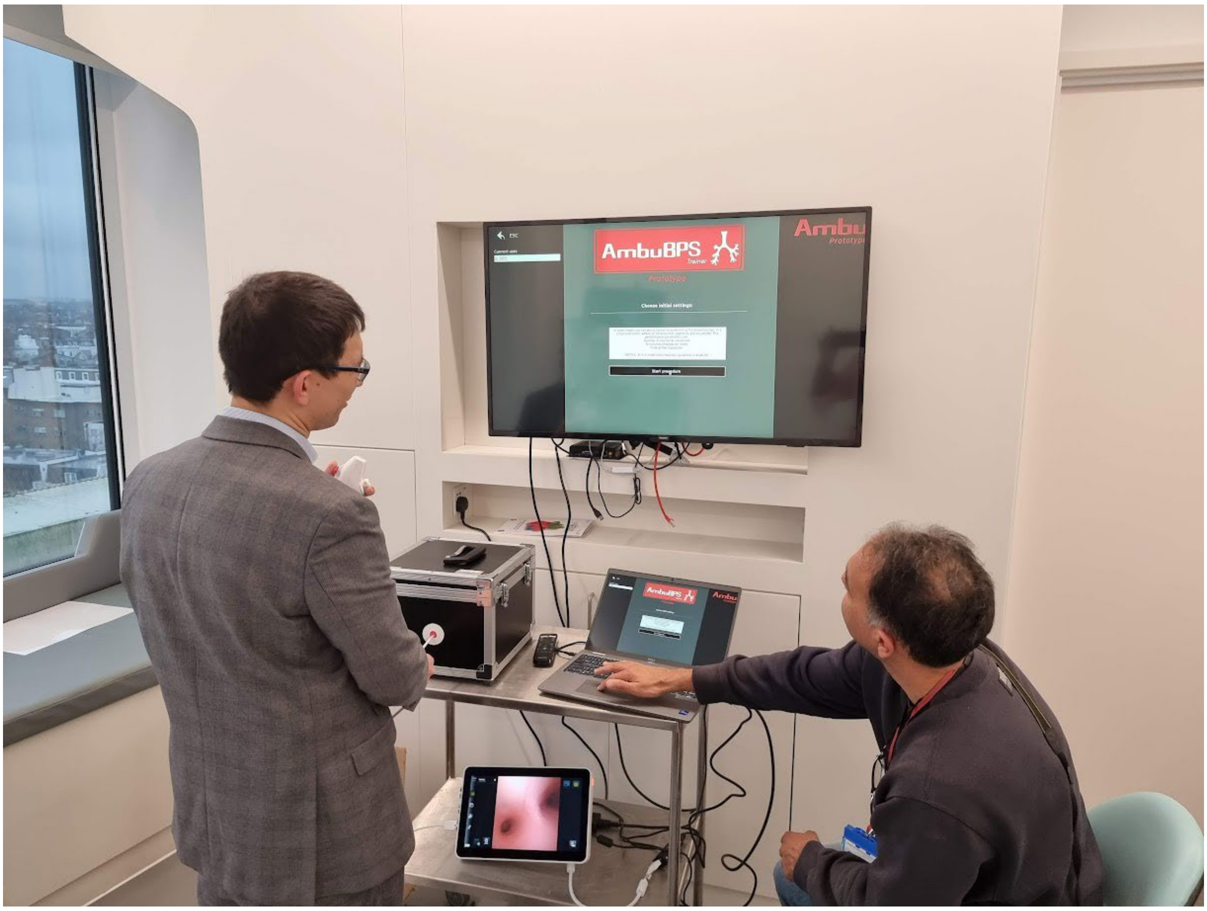

(b) AIG participant undergoing one of her bronchoscopy training runs. Immediate feedback is provided by the system on the screen: Lung tree diagram (bottom right corner), SP score (left side of screen), Augmented Reality label overlays (middle of screen) with "RUL" coloured in green indicating the right upper lobe has been entered (with corresponding dark green colouration on the lung tree diagram). "BI" is still coloured white indicating the bronchus intermedius is yet to be entered (the corresponding light green colouration on the lung tree diagram means it has been visualised on-screen but not yet entered).

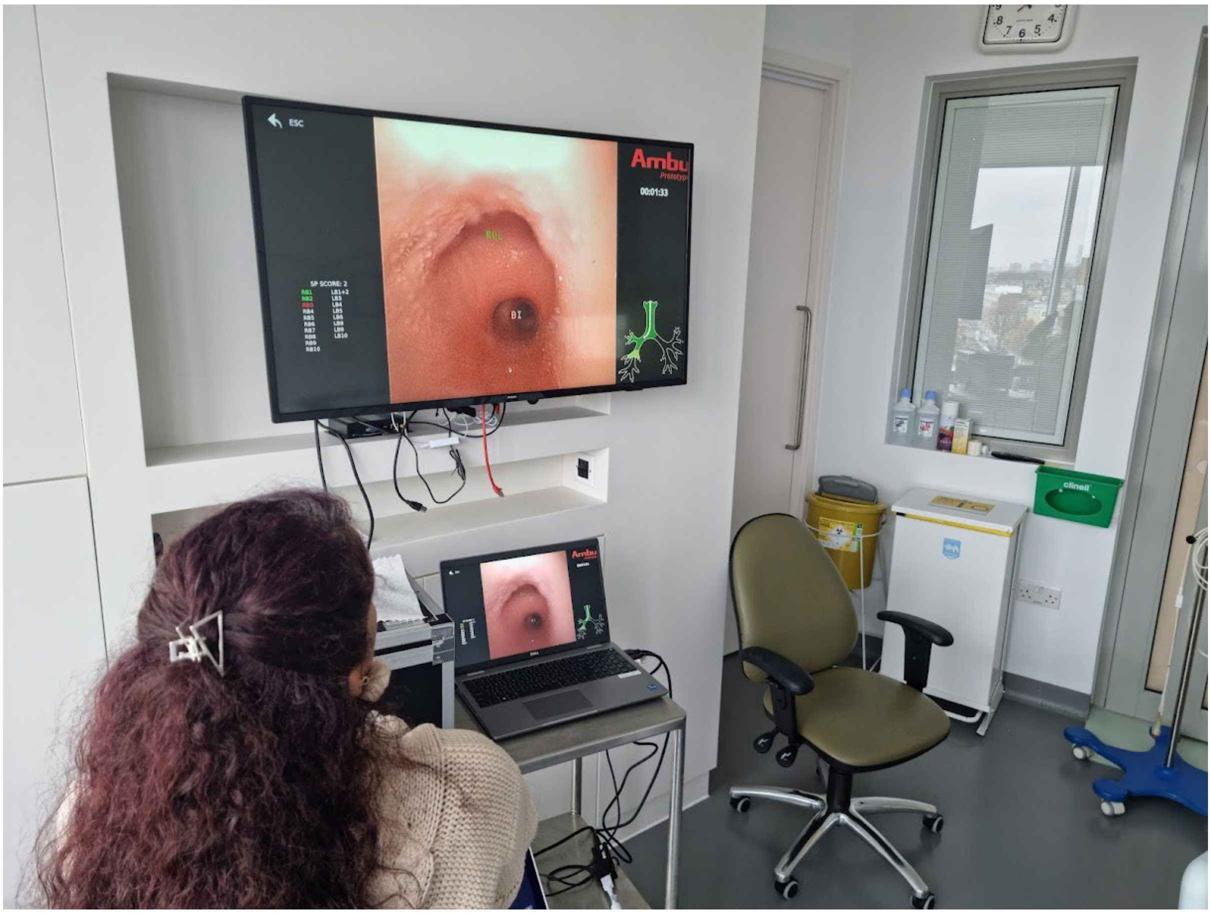

Supplementary Material 4 – Study flow diagram

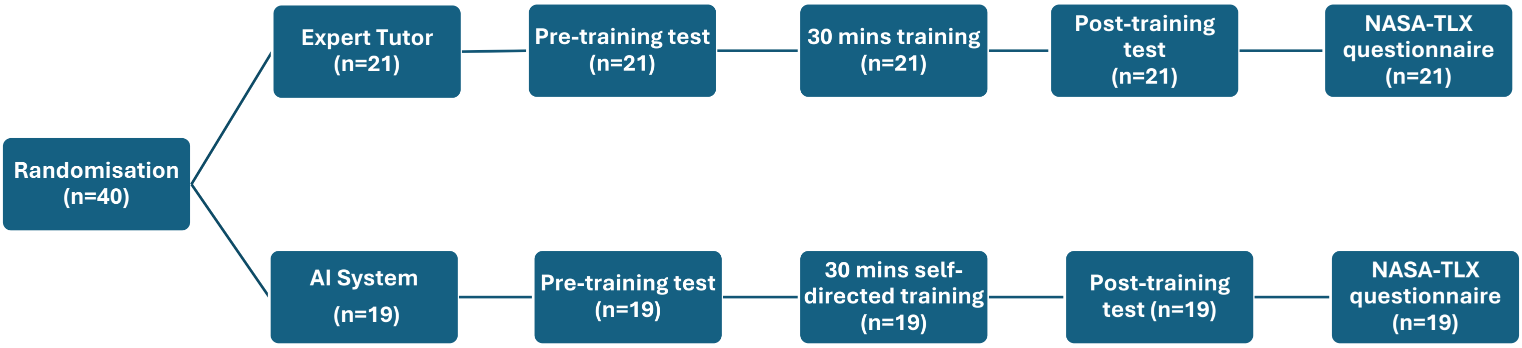

Abbreviations: AI = Artificial Intelligence, NASA-TLX = NASA Task-Load Index

Supplementary Material 5 - NASA-TLX scoring calculation process

Participants were first presented with a series of pairs of rating scale tiles (for example mental demand vs physical demand) (see below) and asked to select which member of each pair contributed more to their experience of workload during the tasks they had just performed (thirty-minutes of training and a final post-training test). Fifteen paired rating scale tiles were presented in total (see below). The number of times that each domain was selected was tallied and used to generate a weighting score for each subscale. For example, if *Performance* was selected five times in the paired tiles, the participant would have a weighting of five for their numerical rating of *Performance* on the rating sheet (see below).

The participants then assign a numerical rating for each subscale by selecting a vertical mark on a dedicated 12-cm horizontal line (see below). Each 12-cm line contains 21 vertical marks, creating a scale with 20 equal intervals. The vertical marks are allocated scores of 0 to 20, left to right, such that if a participant was to select the first vertical mark, a rating of zero would be allocated. If a participant was to mistakenly put their selection between two vertical marks, it is considered as if they had selected the mark on the right (i.e. rounded up).

The overall workload score for each subscale is then generated by multiplying each subscale participant rating by the corresponding weighting score, and then dividing by 15.

NASA-TLX rating scale tiles

Effort<br>or<br>Performance	Temporal Demand<br>or<br>Frustration
Temporal Demand<br>or<br>Effort	Physical Demand<br>or<br>Frustration
Performance<br>or<br>Frustration	Physical Demand<br>or<br>Temporal Demand
Physical Demand<br>or<br>Performance	Temporal Demand<br>or<br>Mental Demand

**Frustration**<br><br>**or**<br><br>**Effort**	**Performance**<br><br>**or**<br><br>**Mental Demand**
**Performance**<br><br>**or**<br><br>**Temporal Demand**	**Mental Demand**<br><br>**or**<br><br>**Effort**
**Mental Demand**<br><br>**or**<br><br>**Physical Demand**	**Effort**<br><br>**or**<br><br>**Physical Demand**
**Frustration**<br><br>**or**<br><br>**Mental Demand**	

NASA-TLX subscale descriptors to aid participant comprehension

Title	Endpoints	Descriptions
MENTAL DEMAND	*Low/High*	How much mental and perceptual activity was required (e.g., thinking, deciding, calculating, remembering, looking, searching, etc.)? Was the task easy or demanding, simple or complex, exacting or forgiving?
PHYSICAL DEMAND	*Low/High*	How much physical activity was required (e.g., pushing, pulling, turning, controlling, activating, etc.)? Was the task easy or demanding, slow or brisk, slack or strenuous, restful or laborious?
TEMPORAL DEMAND	*Low/High*	How much time pressure did you feel due to the rate or pace at which the tasks or task elements occurred? Was the pace slow and leisurely or rapic and frantic?
EFFORT	*Low/High*	How hard did you have to work (mentally and physically) to accomplish your level of performance?
PERFORMANCE	*Good/Poor*	How successful do you think you were in accomplishing the goals of the task set by the experimenter (or yourself)? How satisfied were you with your performance in accomplishing these goals?
FRUSTRATION LEVEL	*Low/High*	How insecure, discouraged, irritated, stressed and annoyed versus secure, gratified, content, relaxed and complacent did you feel during the task?

NASA-TLX rating scale example: the participant would be given a score of 3 for *Mental Demand*

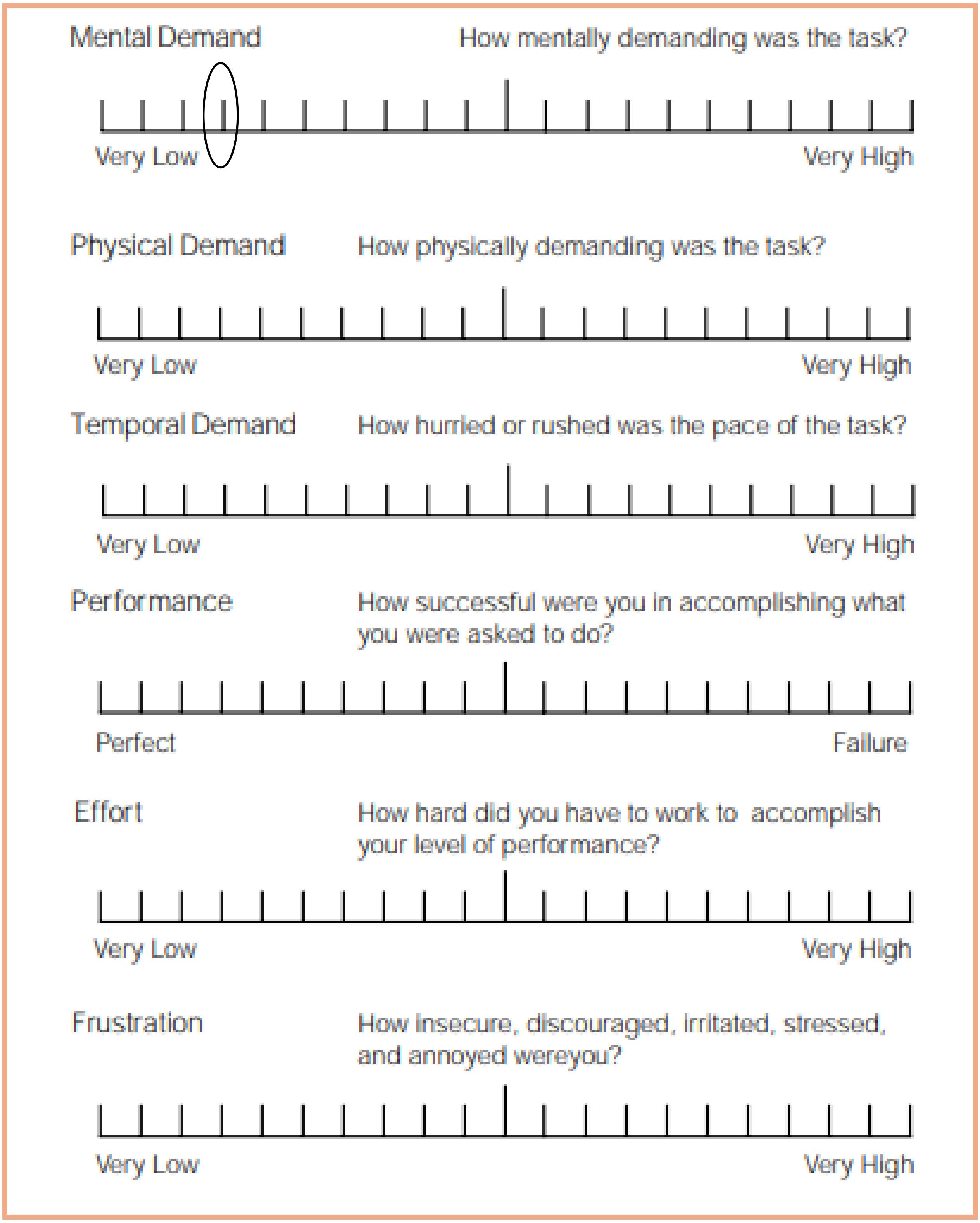

Supplementary Material 6 – Power and Sample size calculations

The standard sample size formula (see below) was used to determine the sample size needed needed per group (AIG and ETG) to give a statistical power of 80%.

The sample size was modelled for different performance metrics. This was based on the primary outcome measure of diagnostic completeness (DC) used in two previous studies (below) - a novice medical student randomised controlled trial[1] and an ICU clinician observational study. We calculated for 80% power to demonstrate a statistically significant difference between arms AIG and ETG at the alpha 0.05 level.

$$N_{per\ group} = \frac{2 \times \bar{\pi} \times (1-\bar{\pi}) \times \left(1.96 + \lambda_{1-\beta}\right)^2}{\left(\pi_{standard} - \pi_{experimental}\right)^2}$$

**Performance outcome measure**	**Medical student study: Control group**	**Medical student study: AI feedback group**	**Pilot clinician: baseline control navigation**	**Pilot clinician: AI feedback navigation**
Procedure Time, PT				
Mean, SD (sec)	434 +/- 172	217+/-127	339+/-150	216+/-150
Diagnostic completion, DC				
Mean, SD (/18 seg)	14.5+/-4	18+/-1	13	15
Structured progress, SP				
Mean, SD	3+/-4	16.5+/-3	4.6	9.4
Mean intersegmental time, MIT				
Mean	30	12	26	22

Table (below) – Estimated sample size needed when attempting to detect significant differences in the different outcome measures.

	**Estimated sample size needed per group***	
**Outcome Measure**	**Medical student study**	**Clinician study**
**PT**	10	24
**DC**	21	16
**SP**	2	11
**MIT**	12	30

* for 80% power and α 0.05.

Based on the above data we modelled the sample size so as to optimise the opportunity for a true effect difference to be identified between AIG and ETG arms within the framework of the study duration. Thus for PT, the sample size was 10-24 per group, DC, the sample size was 16-21 per group. To allow for drop out or non analyzable data, we sought 20 participants per group, i.e., ~40 in total for 80% power with an alpha 0.05 (false positive rate), and 0.2 beta (false negative) limits. For MIT the point effect size difference was 30 or 26 dependent on the study. For an 80% power the current study would require 12-30 per group.

We planned for 20 participants per group for the study.

[1] *Cold KM, Xie S, Nielsen AO, Clementsen PF, Konge L. Artificial intelligence improves novices' bronchoscopy performance: a randomized controlled trial in a simulated setting. Chest. 2024 Feb 1;165(2):405-13.*

Supplementary Material 7 – Table: Comparison of post-training median scores between both groups – outliers included

Metric	ETG (n=21)	AIG (n=19)	Median Difference	P-value
**MIT**	24.4	16.5	**-7.9**	**.027**
**PT**	341	264	**-77**	**.022**
DC	16	16	0	.668
SP	6	6	0	.376
**SR**	14	7	**-7**	**.019**

Post-training bronchoscopy performance scores for MIT, PT, and SR were significantly better in the AIG arm. There were no significant differences in post-training scores for DC and SP.

P-values are calculated using the Mann-Whitney U Test. P values <.05 are considered significant.

Abbreviations: AIG = Artificial Intelligence Group, DC = Diagnostic Completeness (number of segments visited), ETG= Expert Tutor Group, MIT = Mean Intersegmental Time (PT/DC in seconds), PT = Procedure Time (seconds), SR = Segment revisits (number of segments), SP = Structured Progress (points).

Supplementary Material 8 – Table: Comparison of pre-training test median scores between the expert-tutor group (ETG) and artificial-intelligence group (AIG) – outliers included

Metric	ETG (n=21)	AIG (n=19)	P-value
**MIT**	52.3	34.3	**.029**
**PT**	521	380	**.031**
**DC**	10	11	.405
**SP**	2	2	.361
**SR**	14	8	**<.001**

Baseline metrics for MIT, PT, and SR differed despite non-significant differences in group demographics (Table 1). P-values are calculated using the Mann-Whitney U Test. P values <.05 are considered significant.

Abbreviations: DC = Diagnostic Completeness (number of segments visited), MIT = Mean Intersegmental Time (PT/DC in seconds), PT = Procedure Time (seconds), SR = Segment revisits (number of segments), SP = Structured Progress (points).

Supplementary Material 9 – Table: Comparison of pre-training median scores between the expert-tutor group (ETG) and artificial-intelligence group (AIG) – outliers removed

Metric	ETG (n=18)	AIG (n=19)	P-value
**MIT**	51.2	34.3	.105
**PT**	488	380	.126
**DC**	10	11	.298
**SP**	2	2	.258
**SR**	13	4	**<.001**

Baseline metrics for MIT, PT, DC, and SP showed no significant differences. A significant difference in the baseline metric for SR was found despite non-significant differences in group demographics (Table 1). P-values are calculated using the Mann-Whitney U Test. P values <.05 are considered significant.

Abbreviations: DC = Diagnostic Completeness (number of segments visited), MIT = Mean Intersegmental Time (PT/DC in seconds), PT = Procedure Time (seconds), SR = Segment revisits (number of segments), SP = Structured Progress (points).

Supplementary Material 10 – Table: Differences in pre- and post-training performance for both the expert-tutor group (ETG) and artificial-intelligence group (AIG) – outliers removed

Group	Metric	Pre-training	Post-training	P-value
**ETG (n=18)**	**MIT**	51.2	23.9	**.002**
	**PT**	488	338	**.025**
	**DC**	10	15.5	**.001**
	**SP**	2	5.5	**.003**
	SR	13	11.5	.169
**AIG (n=19)**	**MIT**	34.3	16.5	**<.001**
	**PT**	380	264	**<.001**
	**DC**	11	16	**<.001**
	**SP**	2	6	**<.001**
	SR	4	7	**.016**

For both ETG and AIG, the post-training MIT, PT, and DC scores were significantly better. Those in the ETG arm showed no significant difference in SR post-training however, the AIG arm demonstrated a significantly worse SR.

P-values are calculated using the Wilcoxon Signed Rank Test. P values <.05 are considered significant.

Abbreviations: DC = Diagnostic Completeness (number of segments visited), MIT = Mean Intersegmental Time (PT/DC in seconds), PT = Procedure Time (seconds), SR = Segment revisits (number of segments), SP = Structured Progress (points).

Supplementary Material 11 – Figure: Post-training scores between ETG and AIG compared for procedural time (PT) and mean intersegmental time (MIT) – outliers removed

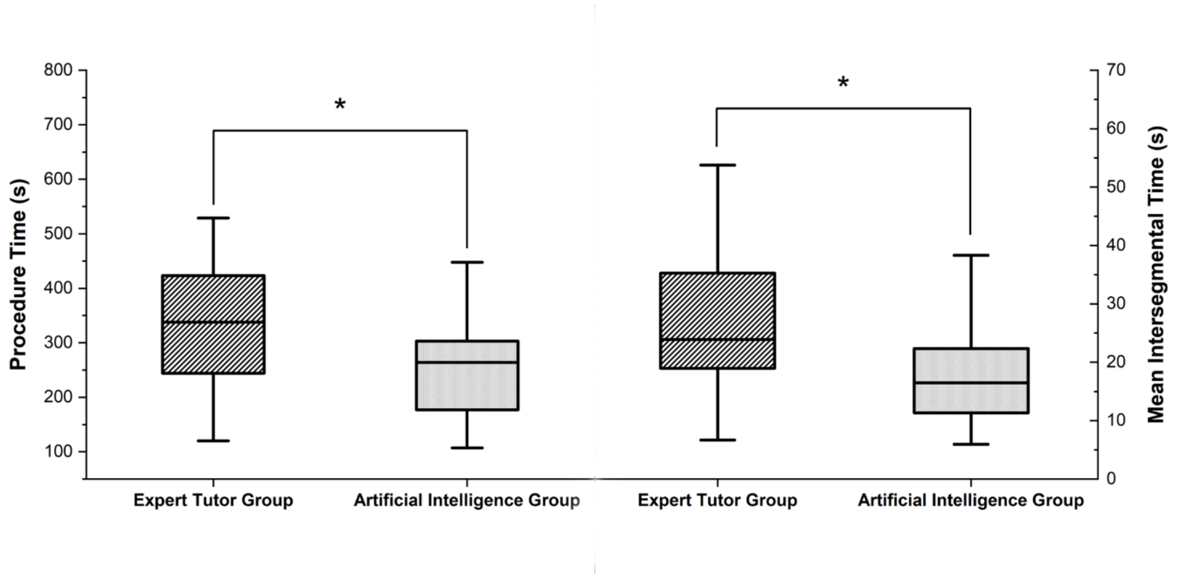

PT: Expert tutor (338 seconds +/- 200), Artificial intelligence (264 seconds +/- 126). MIT: Expert tutor (24 seconds +/- 19), Artificial intelligence (16.5 seconds +/- 11).

Data reported as (median +/- interquartile range). = p < .05 and considered significant.*

Abbreviations: AIG = Artificial Intelligence Group, ETG = Expert Tutor Group, MIT = Mean Intersegmental Time.

Supplementary Material 12 - ETG arm participant allocation numbers and bronchoscopy experience – outliers included

Participant Number	Pre-training					Post-training				
	MIT	PT	DC	SP	SR	MIT	PT	DC	SP	SR
**p_002**	86.6	606	7	2	8	81.1	892	11	1	18
**p_005**	14.4	216	15	11	13	19.5	293	15	13	6
**p_006**	50.2	502	10	4	7	15.1	271	18	13	11
**p_008**	38.2	382	10	3	8	24.4	244	10	2	2
**p_010**	33	264	8	2	13	53.8	215	4	1	5
**p_014**	52.3	471	9	0	17	22.3	335	15	3	12
**p_015**	52.2	522	10	0	2	35.3	529	15	3	12
**p_016**	77.4	697	9	1	23	18.9	322	17	5	13
**p_019**	31.8	414	13	0	14	10.1	182	18	10	4
**p_023**	147	1179	8	5	14	81.1	1297	16	5	34
**p_024**	43.1	474	11	5	14	53.6	858	16	4	46
**p_027**	84.7	847	10	0	33	23.4	398	17	6	13

**p_028**	57.9	405	7	2	2	42.1	379	9	6	6
**p_029**	31.6	443	14	3	11	20.1	341	17	9	10
**p_030**	44.9	629	14	0	40	28	476	17	2	35
**p_031**	75.6	1209	16	1	65	16.4	263	16	12	10
**p_034**	59.4	594	10	6	21	35.3	423	12	6	13
**p_035**	82.4	1154	14	6	22	27.6	441	16	6	4
**p_036**	57.9	521	9	3	6	13.2	211	16	10	2
**p_037**	46.2	277	6	1	6	6.67	120	18	17	2
**p_039**	55	770	14	2	30	25.3	379	15	2	21

Outliers, participants p_023, p_031, p_035, are shaded in grey.

Abbreviations: DC = Diagnostic Completeness (number of segments visited), ETG = Expert Tutor Group, MIT = Mean Intersegmental Time (PT/DC in seconds), PT = Procedure Time (seconds), SR = Segment revisits (number of segments), SP = Structured Progress (points).

Supplementary Material 13 – AIG arm participant allocation numbers and bronchoscopy experience – outliers included

Participant Number	Pre-training					Post-training				
	MIT	PT	DC	SP	SR	MIT	PT	DC	SP	SR
**p_001**	14.1	239	17	12	1	5.94	107	18	18	0
**p_003**	33.7	236	7	0	2	16.1	177	11	4	2
**p_004**	22.1	199	9	1	1	6.75	108	16	11	0
**p_007**	21.2	233	11	2	0	20.3	264	13	4	10
**p_009**	63.3	759	12	2	8	21.6	303	14	6	14
**p_011**	26.7	454	17	6	9	7.39	133	18	16	2
**p_012**	25.3	380	15	5	4	13.9	250	18	15	12
**p_013**	62.3	374	6	3	2	27.5	303	11	3	8
**p_017**	100	802	8	1	5	26.7	240	9	1	15
**p_018**	58.9	589	10	1	6	38.3	575	15	1	24
**P_020**	40.3	403	10	2	6	22.4	380	17	4	23

**p_021**	38.3	230	6	0	2	21.9	285	13	4	8
**p_022**	16.6	283	17	9	4	11.4	193	17	12	5
**p_025**	34.3	548	16	7	5	21	273	13	6	7
**p_026**	42.9	472	11	1	3	16	288	18	10	6
**p_032**	59.2	710	12	4	8	29.9	448	15	4	19
**p_033**	54	540	10	5	1	16.5	297	18	7	0
**p_038**	15.4	216	14	8	2	12.6	215	17	13	4
**p_040**	28.9	289	10	2	9	7.06	120	17	13	2

Abbreviations: AIG = Artificial Intelligence Group, DC = Diagnostic Completeness (number of segments visited), MIT = Mean Intersegmental Time (PT/DC in seconds), PT = Procedure Time (seconds), SR = Segment revisits (number of segments), SP = Structured Progress (points).

Supplementary Material 14 – Individual pre- and post-training scores for both the ETG and AIG arms – outliers included

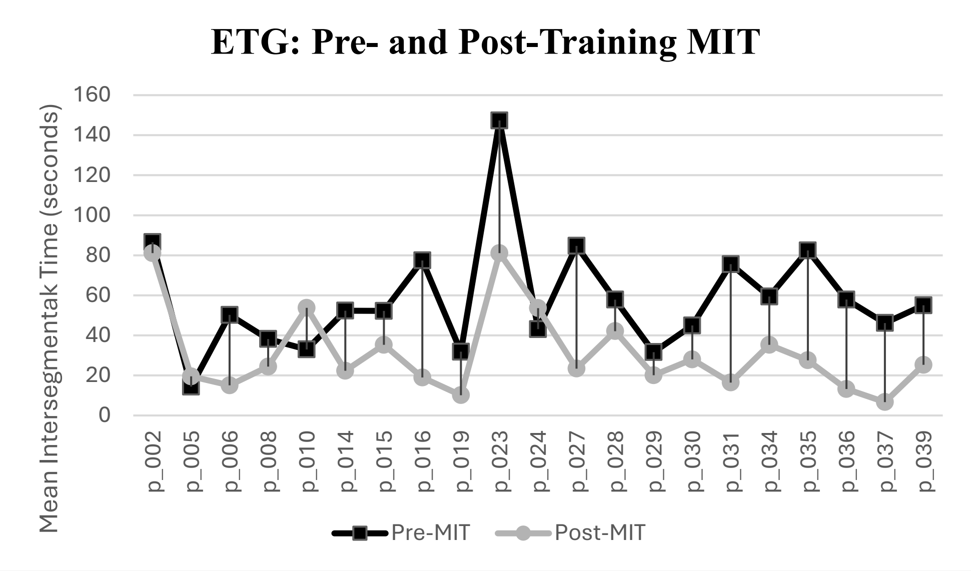

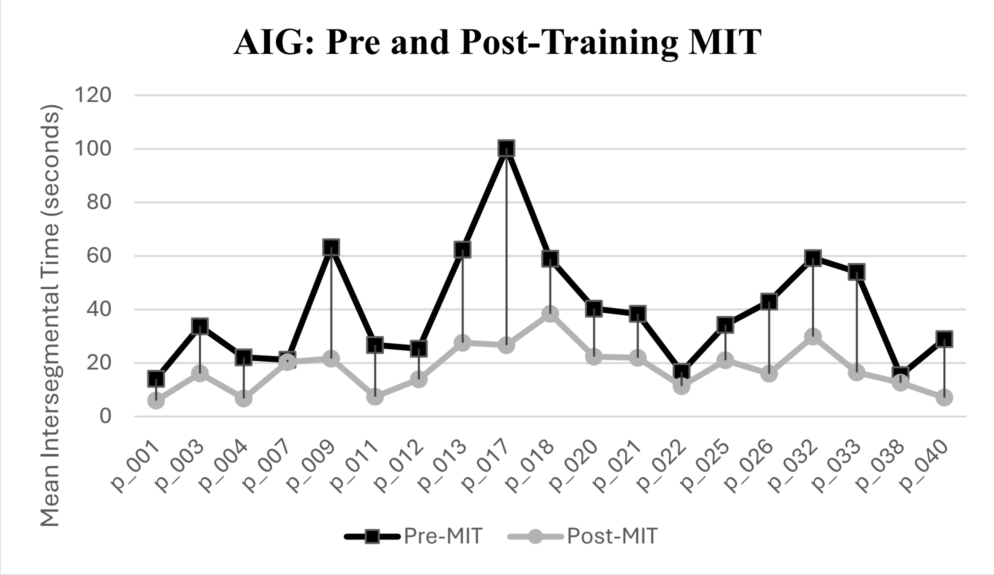

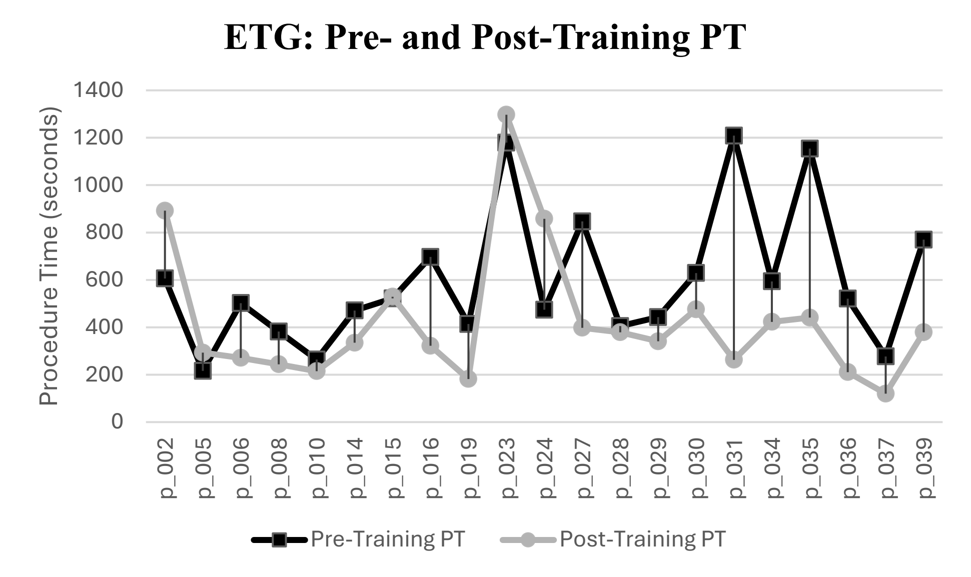

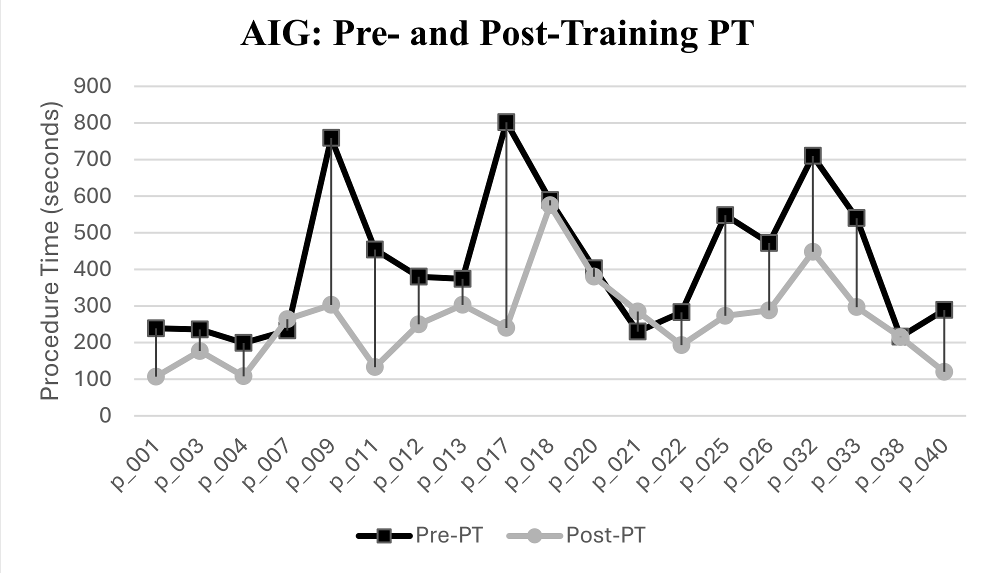

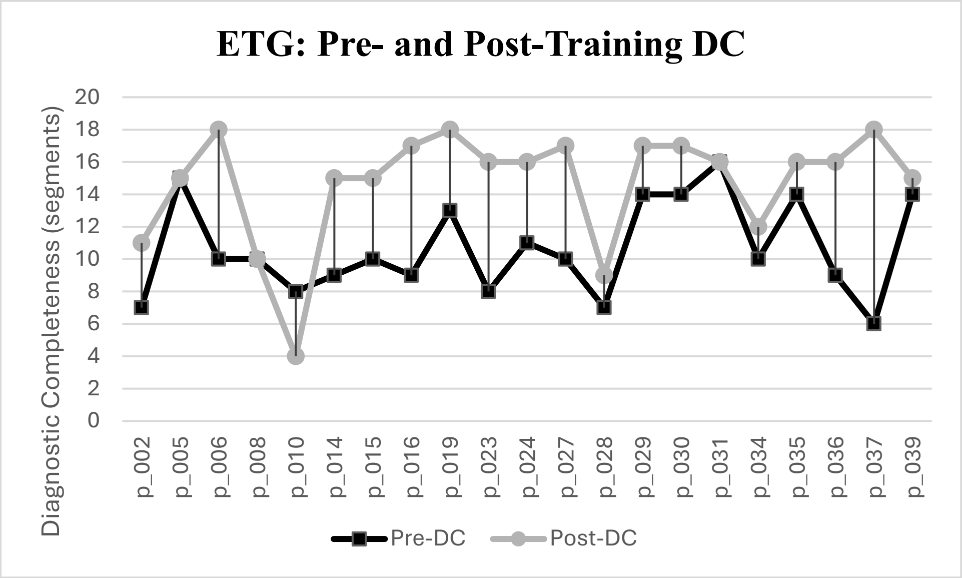
ETG: Pre- and Post-Training DC
Diagnostic Completeness (segments)
p_002 p_005 p_006 p_008 p_010 p_014 p_015 p_016 p_019 p_023 p_024 p_027 p_028 p_029 p_030 p_031 p_034 p_035 p_036 p_037 p_039
Pre-DC
Post-DC

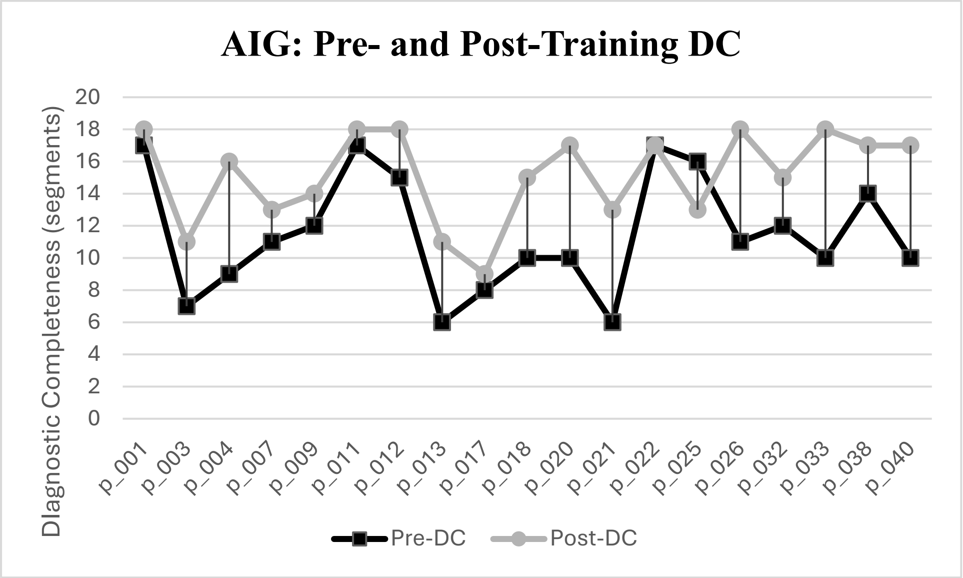
AIG: Pre- and Post-Training DC
Diagnostic Completeness (segments)
p_001 p_003 p_004 p_007 p_009 p_011 p_012 p_013 p_017 p_018 p_020 p_021 p_022 p_025 p_026 p_032 p_033 p_038 p_040
Pre-DC
Post-DC

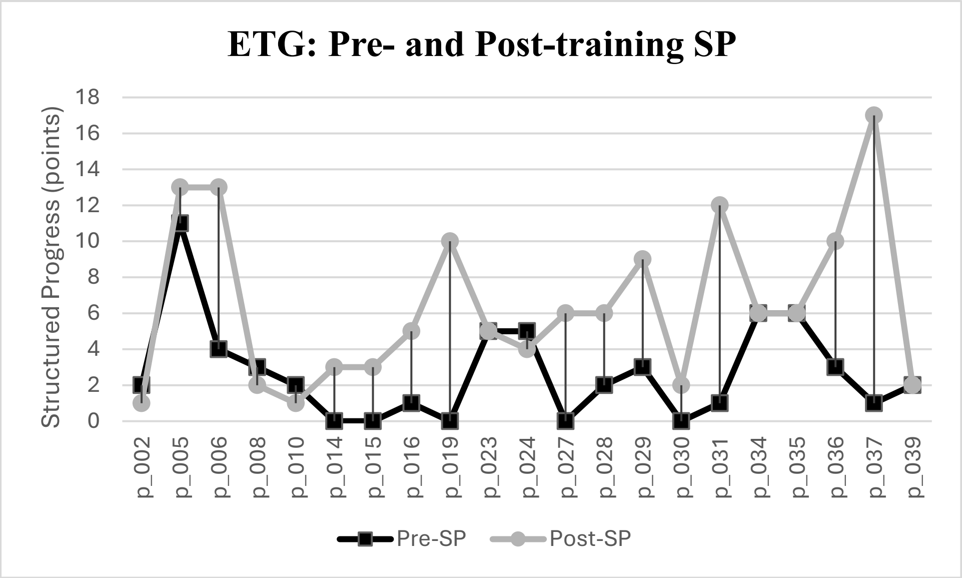
ETG: Pre- and Post-training SP
Structured Progress (points)
p_002 p_005 p_006 p_008 p_010 p_014 p_015 p_016 p_019 p_023 p_024 p_027 p_028 p_029 p_030 p_031 p_034 p_035 p_036 p_037 p_039
Pre-SP
Post-SP

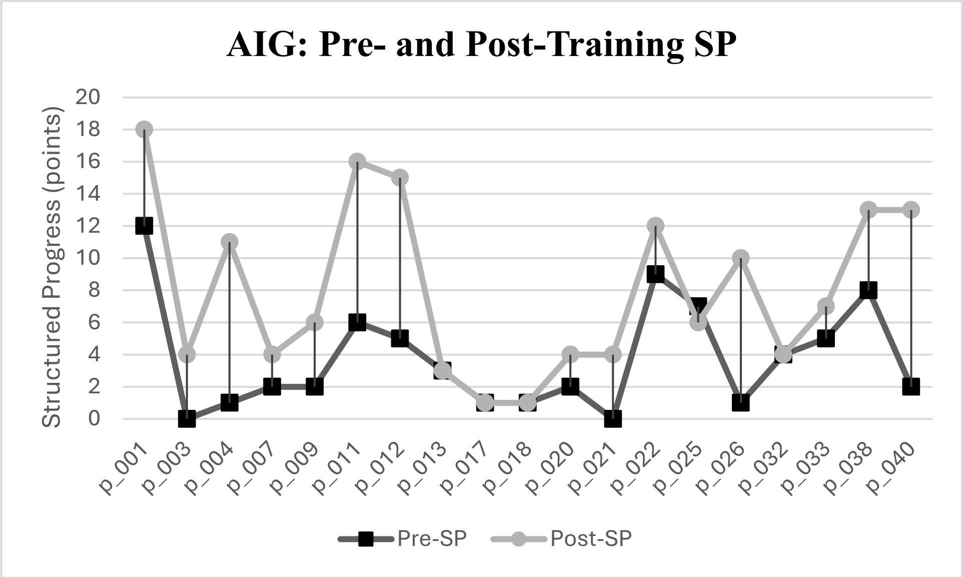
AIG: Pre- and Post-Training SP
Structured Progress (points)
p_001 p_003 p_004 p_007 p_009 p_011 p_012 p_013 p_017 p_018 p_020 p_021 p_022 p_025 p_026 p_032 p_033 p_038 p_040
Pre-SP
Post-SP

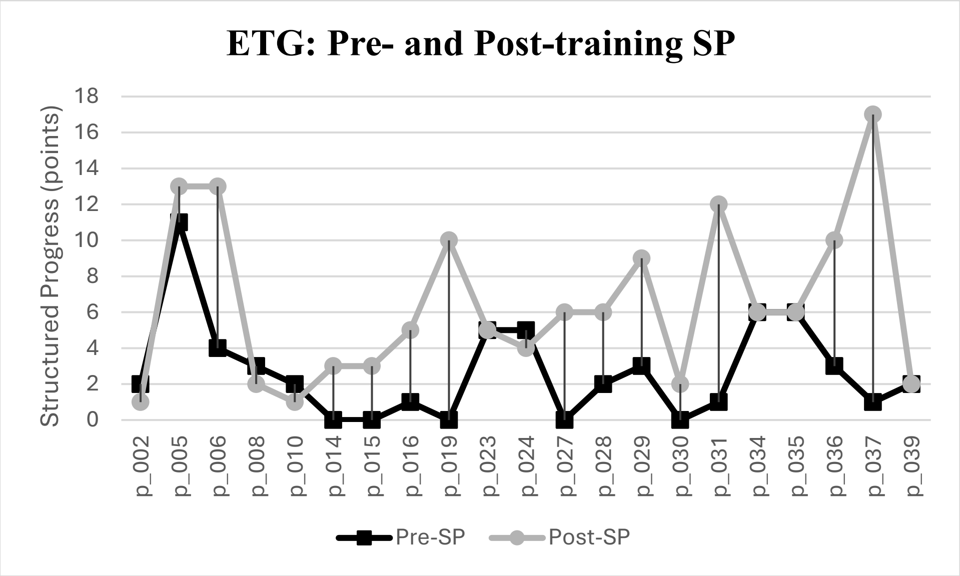
ETG: Pre- and Post-training SP
Structured Progress (points)
0 2 4 6 8 10 12 14 16 18
p_002 p_005 p_006 p_008 p_010 p_014 p_015 p_016 p_019 p_023 p_024 p_027 p_028 p_029 p_030 p_031 p_034 p_035 p_036 p_037 p_039
Pre-SP
Post-SP

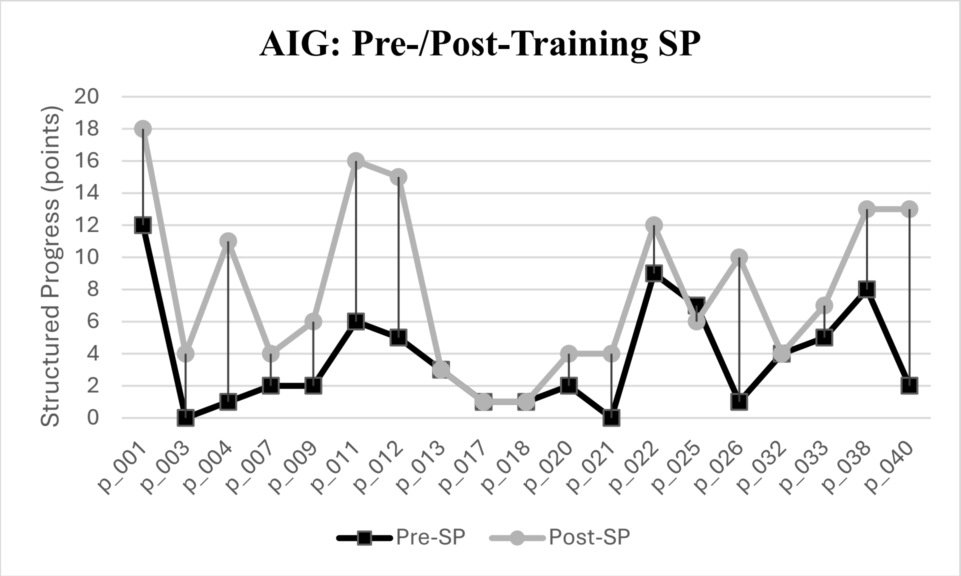
AIG: Pre-/Post-Training SP
Structured Progress (points)
0 2 4 6 8 10 12 14 16 18 20
p_001 p_003 p_004 p_007 p_009 p_011 p_012 p_013 p_017 p_018 p_020 p_021 p_022 p_025 p_026 p_032 p_033 p_038 p_040
Pre-SP
Post-SP

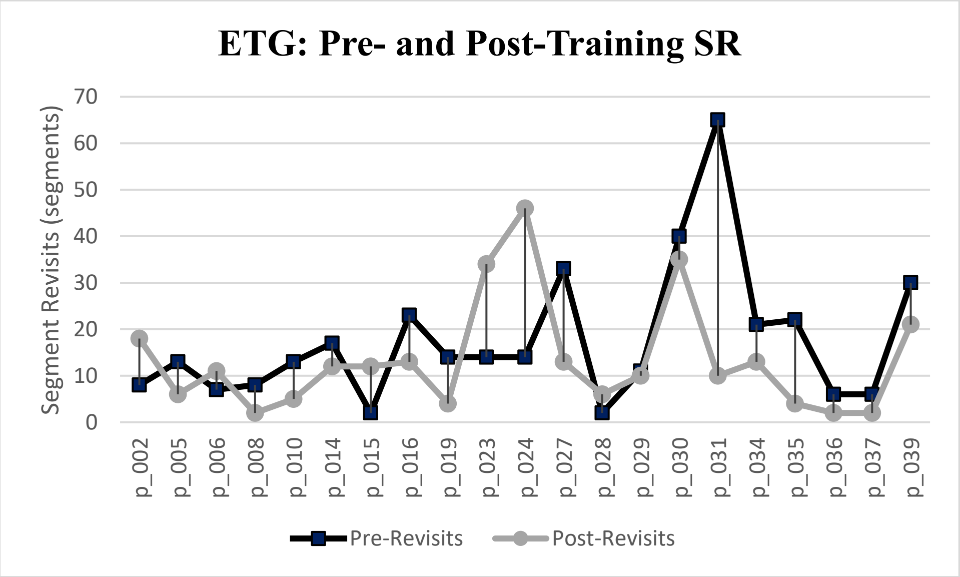
ETG: Pre- and Post-Training SR
Segment Revisits (segments)
0 10 20 30 40 50 60 70
p_002 p_005 p_006 p_008 p_010 p_014 p_015 p_016 p_019 p_023 p_024 p_027 p_028 p_029 p_030 p_031 p_034 p_035 p_036 p_037 p_039
Pre-Revisits
Post-Revisits

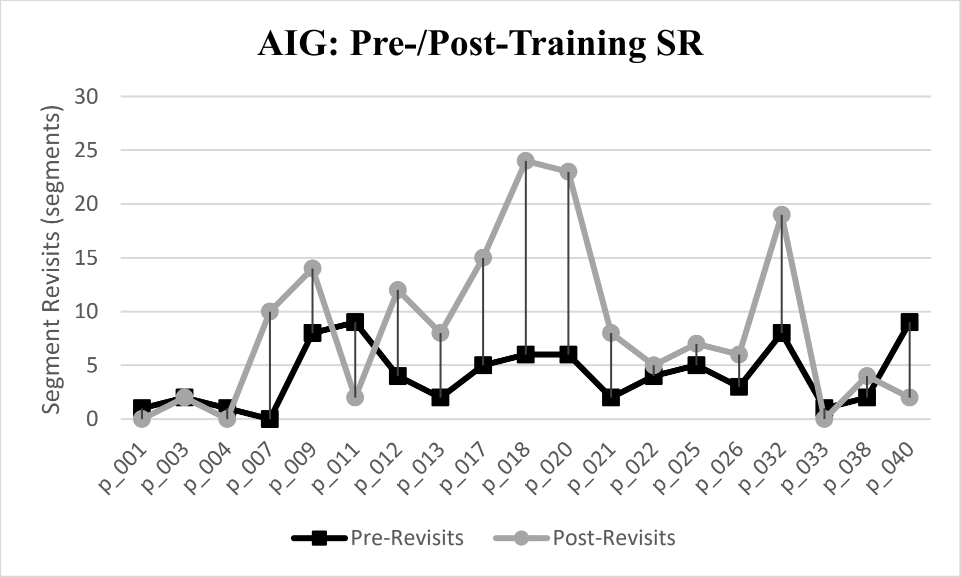
AIG: Pre-/Post-Training SR
Segment Revisits (segments)
0 5 10 15 20 25 30
p_001 p_003 p_004 p_007 p_009 p_011 p_012 p_013 p_017 p_018 p_020 p_021 p_022 p_025 p_026 p_032 p_033 p_038 p_040
Pre-Revisits
Post-Revisits

Line charts demonstrating pre- and post-training bronchoscopy performance scores in each analysed metric for individual participants in the ETG and AIG arms. Vertical lines connecting data points join pairs of data points for the same participant.

Abbreviations: AIG = Artificial Intelligence Group, DC = Diagnostic Completeness (number of segments visited), ETG = Expert Tutor Group, MIT = Mean Intersegmental Time (PT/DC in seconds), PT = Procedure Time (seconds), SR = Segment revisits (number of segments), SP = Structured Progress (points).

Supplementary Material 15 – ETG arm participant bronchoscopy experience

Participant Number	Physician Grade	Experience Level	Total No. of Bronchoscopies	No. of bronchoscopies Last 6 Months
**p_002**	**Senior Trainee**	**Intermediate**	17	2
**p_005**	**Attending**	**Expert**	1000	400
**p_006**	**Senior Trainee**	**Intermediate**	16	3
**p_008**	**Senior Trainee**	**Novice**	1	1
**p_010**	**Attending**	**Intermediate**	50	10
**p_014**	**Senior Trainee**	**Novice**	2	2
**p_015**	**Senior Trainee**	**Expert**	100	15
**p_016**	**Senior Trainee**	**Intermediate**	14	4
**p_019**	**Attending**	**Intermediate**	50	4
**p_023**	**Senior Trainee**	**Novice**	2	0
**p_024**	**Junior Trainee**	**Intermediate**	10	5
**p_027**	**Junior Trainee**	**Novice**	5	5
**p_028**	**Senior Trainee**	**Intermediate**	40	0
**p_029**	**Attending**	**Expert**	150	10
**p_030**	**Junior Trainee**	**Intermediate**	38	5
**p_031**	**Senior Trainee**	**Intermediate**	16	3
**p_034**	**Senior Trainee**	**Intermediate**	30	10
**p_035**	**Senior Trainee**	**Novice**	2	0
**p_036**	**Junior Trainee**	**Novice**	2	2
**p_037**	**Senior Trainee**	**Intermediate**	20	5
**p_039**	**Senior Trainee**	**Intermediate**	12	5

Abbreviations: ETG = Expert Tutor Group

Supplementary Material 16 – AIG arm participant bronchoscopy experience

Participant Number	Physician Grade	Experience Level	Total No. of Bronchoscopies	No. of bronchoscopies Last 6 Months
p_001	Attending	Expert	400	35
p_003	Junior Trainee	Novice	3	3
p_004	Senior Trainee	Intermediate	20	4
p_007	Attending	Intermediate	15	0
p_009	Attending	Expert	1000	1
p_011	Junior Trainee	Intermediate	50	20
p_012	Attending	Intermediate	29	1
p_013	Senior Trainee	Intermediate	30	0
p_017	Junior Trainee	Novice	2	2
p_018	Junior Trainee	Novice	2	2
P_020	Junior Trainee	Novice	2	0
p_021	Senior Trainee	Intermediate	20	3
p_022	Senior Trainee	Intermediate	50	15
p_025	Senior Trainee	Intermediate	15	5
p_026	Junior Trainee	Novice	1	1
p_032	Senior Trainee	Intermediate	20	5
p_033	Attending	Expert	100	15
p_038	Attending	Expert	100	8
p_040	Junior Trainee	Novice	2	0

Abbreviations: AIG = Artificial Intelligence Group

Supplementary Material 17 – Table: NASA-TLX cognitive workload scores between the expert-tutor group (ETG) and artificial-intelligence group (AIG) – outliers included

Workload Domain	ETG (n =21)	AIG (n =19)	Median Difference	P-value
**Mental Demand**	56	42	-14	.270
**Physical Demand**	6	8	+2	.649
**Temporal Demand**	21	26	+5	.333
**Performance**	39	36	+6	.979
**Effort**	30	27	-3	.630
**Frustration**	2	0	-2	.915
**Overall Perceived Workload**	178	179	+1	.915

No significant difference was found between the scores in any domains for both groups.

P values <.05 are considered significant.

P-values were calculated using the Mann-Whitney U test. P values <.05 are considered significant. Abbreviations: AIG = Artificial Intelligence Group, ETG = Expert Tutor Group, NASA-TLX = NASA Task Load Index.

Supplementary Material 18 – NASA-TLX cognitive workload score comparison between the expert-tutor group (ETG) and artificial-intelligence group (AIG) – outliers removed

Workload Domain	ETG (n=18)	AIG (n=19)	Median Difference	P-value
**Mental Demand**	43.5	42	-1.5	.578
**Physical Demand**	5.5	8	+2.5	.730
**Temporal Demand**	22.5	26	+3.5	.358
**Performance**	35.5	36	+0.5	.916
**Effort**	29	27	-2	.578
**Frustration**	1	0	-1	.707
**Overall Perceived Workload**	173	179	+6	.538

No significant differences were found between the scores in any domains for both groups.

P-values were calculated using the Mann-Whitney U test. P values <.05 are considered significant.

Abbreviations: AIG = Artificial Intelligence Group, ETG = Expert Teacher Group.
